# 2-(5-Chloro-1,3-benzothia­zol-2-yl)-4-meth­oxy­phenol

**DOI:** 10.1107/S1600536812037804

**Published:** 2012-09-08

**Authors:** Sammer Yousuf, Shazia Shah, Nida Ambreen, Khalid M. Khan, Shakil Ahmad

**Affiliations:** a H.E.J. Research Institute of Chemistry, International Center for Chemical and Biological Sciences, University of Karachi, Karachi 75270, Pakistan

## Abstract

In the mol­ecule of the title compound, C_14_H_10_ClNO_2_S, the dihedral angle between the almost planar benzothia­zole ring system [maximum deviation = 0.005 (2) Å] and the benzene ring is 1.23 (9)°. The conformation of the mol­ecule is stabilized by an intra­molecular O—H⋯N hydrogen bond, forming an *S*(6) ring motif. In the crystal, mol­ecules are linked into layers parallel to the *ac* plane by C—H⋯O hydrogen bonds and π–π stacking inter­actions [centroid–centroid distance = 3.7365 (12) Å].

## Related literature

For the biological activity of benzothia­zole compounds see: Sreenivasa *et al.* (2009 ▶); Maharan *et al.* (2007 ▶); Pattan *et al.* (2005 ▶); Chohan *et al.* (2003 ▶); Bénéteau *et al.* (1999 ▶). For the crystal structures of benzothia­zole derivatives, see: Lakshmanan *et al.* (2011 ▶); Zhang *et al.* (2008 ▶).
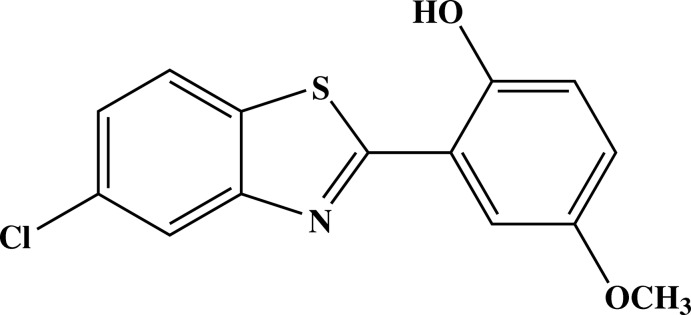


## Experimental

### 

#### Crystal data

C_14_H_10_ClNO_2_S
*M* *_r_* = 291.74
Orthorhombic, 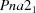

*a* = 7.4877 (4) Å
*b* = 27.2166 (15) Å
*c* = 6.1902 (3) Å
*V* = 1261.50 (11) Å^3^
*Z* = 4
Mo *K*α radiation
μ = 0.46 mm^−1^
*T* = 273 K
0.38 × 0.25 × 0.12 mm

#### Data collection

Bruker SMART APEX CCD area-detector diffractometer
Absorption correction: multi-scan (*SADABS*; Bruker, 2000 ▶) *T*_min_ = 0.844, *T*_max_ = 0.947
7114 measured reflections
2226 independent reflections
2134 reflections with *I* > 2σ(*I*)
*R*_int_ = 0.018

#### Refinement

*R*[*F* ^2^ > 2σ(*F* ^2^)] = 0.026
*wR*(*F* ^2^) = 0.069
*S* = 1.07
2226 reflections
177 parameters
1 restraint
H atoms treated by a mixture of independent and constrained refinement
Δρ_max_ = 0.16 e Å^−3^
Δρ_min_ = −0.17 e Å^−3^
Absolute structure: Flack (1983 ▶), 935 Friedel pairs
Flack parameter: 0.07 (6)

### 

Data collection: *SMART* (Bruker, 2000 ▶); cell refinement: *SAINT* (Bruker, 2000 ▶); data reduction: *SAINT*; program(s) used to solve structure: *SHELXS97* (Sheldrick, 2008 ▶); program(s) used to refine structure: *SHELXL97* (Sheldrick, 2008 ▶); molecular graphics: *SHELXTL* (Sheldrick, 2008 ▶); software used to prepare material for publication: *SHELXTL*, *PARST* (Nardelli, 1995 ▶) and *PLATON* (Spek, 2009 ▶).

## Supplementary Material

Crystal structure: contains datablock(s) global, I. DOI: 10.1107/S1600536812037804/rz5002sup1.cif

Structure factors: contains datablock(s) I. DOI: 10.1107/S1600536812037804/rz5002Isup2.hkl

Supplementary material file. DOI: 10.1107/S1600536812037804/rz5002Isup3.cml

Additional supplementary materials:  crystallographic information; 3D view; checkCIF report

## Figures and Tables

**Table 1:** Hydrogen-bond geometry (Å, °)

*D*—H⋯*A*	*D*—H	H⋯*A*	*D*⋯*A*	*D*—H⋯*A*
O1—H1*A*⋯N1	0.80 (3)	1.87 (3)	2.612 (2)	154 (3)
C5—H5*A*⋯O2^i^	0.93	2.57	3.454 (3)	159

Symmetry code: (i) 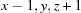.

**Fig. 1.**
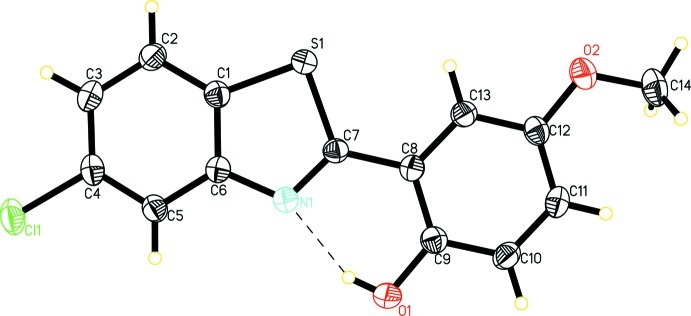
The molecular structure of title compound with displacement ellipsoids drawn at 30% probability level. The intramolecular hydrogen bond is shown as a dashed line.

**Fig. 2.**
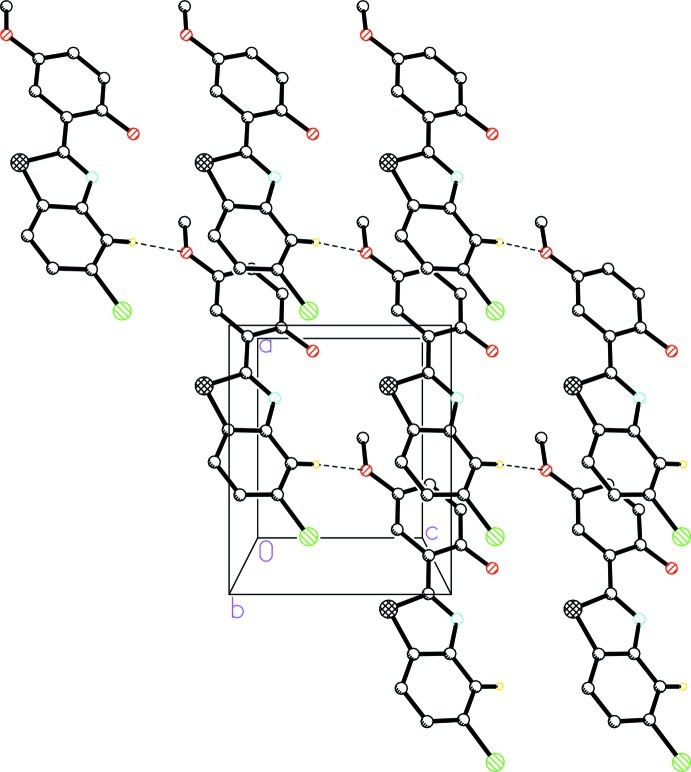
The crystal packing of the title compound. Hydrogen atoms not involved in intermolecular hydrogen bonds (dashed lines) are omitted.

## supplementary crystallographic information

### Comment

The heterocyclic compounds containing the benzothiazole moeity as basic skeleton are well known to have a broad range of biological properties (Sreenivasa *et al.*, 2009; Maharan *et al.*, 2007; Pattan *et al.*, 2005; Chohan *et al.*, 2003; Bénéteau *et al.*, 1999). The title compound is a benzothiazole derivative synthesized in order to study the different biological activities of these compounds. In the title compound (Fig. 1) the chloro susbtitued benzothiazole (S1/N1/C1–C7) and methoxy substituted phenol rings (C8–C13) are each planar with maximum devaiation of -0.005 (2) Å for atom C1. The dihedral angle between them is 1.23 (9)°. All bond lengths are in agreement with those found in related benzothiazole structures (Lakshmanan *et al.*, 2011; Zhang *et al.*, 2008). The C5—H5A···O2 hydrogen bonds play an important role in stabilizing the crystal structure by forming a two-dimensional network (symmetry codes as in Table 1, Fig. 2) which is further strengthened by significant π..π interactions between phenyl rings (*Cg1*···*Cg2*^i^ = 3.7365 (12) Å;*Cg1* and *Cg2* are the centroids of the C1–C6 and C8–C13 rings, respectively; symmetry code: (i) -1+x, y, z).

### Experimental

In a 50 ml round-bottomed flask 2-amino-4-cholorobenzenethiol (0.159 g, 1 mmol), 2-hydroxy-5-methoxybenzaldehyde (0.152 g, 1 mmol), *N*,*N*-dimethylformamide (10 ml) and sodium metabisulfite (0.2 g) were added with continuous stirring. The reaction mixture was refluxed for 2 h and the progress of the reaction was monitored by TLC. After completion of the reaction, the mixture was allowed to cool at room temperature and addition of cold water produced a solid precipitate. Crystallization from ethanol afforded crystal of 2-(5-chloro-1,3-benzothiazol-2-yl)-4-methoxyphenol (0.235 g, 80.8% yield) which were found suitable for single crystal X-ray diffraction studies.

### Refinement

H atoms of methyl and phenyl carbon atoms were positioned geometrically with 0.96 and 0.93 Å, respectively, and constrained to ride on their parent atoms with *U*_iso_(H) = 1.2*U*_eq_(CH_2_) or 1.5*U*_eq_(CH_3_). A rotating group model was applied to the methyl group.

### Crystal data

**Table:**

C_14_H_10_ClNO_2_S	*F*(000) = 600
*M**_r_* = 291.74	*D*_x_ = 1.536 Mg m^−^^3^
Orthorhombic, *P**n**a*2_1_	Mo *K*α radiation, λ = 0.71073 Å
Hall symbol: P 2c -2n	Cell parameters from 3878 reflections
*a* = 7.4877 (4) Å	θ = 3.0–27.6°
*b* = 27.2166 (15) Å	µ = 0.46 mm^−^^1^
*c* = 6.1902 (3) Å	*T* = 273 K
*V* = 1261.50 (11) Å^3^	Block, colorles
*Z* = 4	0.38 × 0.25 × 0.12 mm

### Data collection

**Table:**

Bruker SMART APEX CCD area-detector diffractometer	2226 independent reflections
Radiation source: fine-focus sealed tube	2134 reflections with *I* > 2σ(*I*)
Graphite monochromator	*R*_int_ = 0.018
ω scan	θ_max_ = 25.5°, θ_min_ = 1.5°
Absorption correction: multi-scan (*SADABS*; Bruker, 2000)	*h* = −9→9
*T*_min_ = 0.844, *T*_max_ = 0.947	*k* = −31→32
7114 measured reflections	*l* = −7→7

### Refinement

**Table:**

Refinement on *F*^2^	Secondary atom site location: difference Fourier map
Least-squares matrix: full	Hydrogen site location: inferred from neighbouring sites
*R*[*F*^2^ > 2σ(*F*^2^)] = 0.026	H atoms treated by a mixture of independent and constrained refinement
*wR*(*F*^2^) = 0.069	*w* = 1/[σ^2^(*F*_o_^2^) + (0.0397*P*)^2^ + 0.1635*P*] where *P* = (*F*_o_^2^ + 2*F*_c_^2^)/3
*S* = 1.07	(Δ/σ)_max_ < 0.001
2226 reflections	Δρ_max_ = 0.16 e Å^−^^3^
177 parameters	Δρ_min_ = −0.17 e Å^−^^3^
1 restraint	Absolute structure: Flack (1983), 935 Friedel pairs
Primary atom site location: structure-invariant direct methods	Flack parameter: 0.07 (6)

### Special details

**Table:**

Geometry. All e.s.d.'s (except the e.s.d. in the dihedral angle between two l.s. planes) are estimated using the full covariance matrix. The cell e.s.d.'s are taken into account individually in the estimation of e.s.d.'s in distances, angles and torsion angles; correlations between e.s.d.'s in cell parameters are only used when they are defined by crystal symmetry. An approximate (isotropic) treatment of cell e.s.d.'s is used for estimating e.s.d.'s involving l.s. planes.
Refinement. Refinement of *F*^2^ against ALL reflections. The weighted *R*-factor *wR* and goodness of fit *S* are based on *F*^2^, conventional *R*-factors *R* are based on *F*, with *F* set to zero for negative *F*^2^. The threshold expression of *F*^2^ > σ(*F*^2^) is used only for calculating *R*-factors(gt) *etc*. and is not relevant to the choice of reflections for refinement. *R*-factors based on *F*^2^ are statistically about twice as large as those based on *F*, and *R*- factors based on ALL data will be even larger.

### Fractional atomic coordinates and isotropic or equivalent isotropic displacement parameters (Å^2^)

**Table:**

	*x*	*y*	*z*	*U*_iso_*/*U*_eq_	
S1	0.76761 (6)	0.412840 (19)	0.76293 (9)	0.04557 (14)	
Cl1	0.10003 (7)	0.45023 (2)	1.33138 (13)	0.06375 (18)	
O1	0.9266 (2)	0.31641 (6)	1.3478 (3)	0.0566 (4)	
H1A	0.842 (4)	0.3333 (10)	1.319 (5)	0.070 (9)*	
O2	1.3785 (2)	0.32630 (6)	0.6457 (3)	0.0618 (4)	
N1	0.7107 (2)	0.37543 (6)	1.1415 (3)	0.0409 (4)	
C1	0.5714 (2)	0.42859 (7)	0.8955 (3)	0.0414 (5)	
C2	0.4317 (2)	0.45863 (7)	0.8272 (4)	0.0474 (5)	
H2A	0.4353	0.4742	0.6935	0.057*	
C3	0.2873 (3)	0.46464 (8)	0.9648 (4)	0.0494 (5)	
H3A	0.1917	0.4843	0.9233	0.059*	
C4	0.2850 (3)	0.44153 (7)	1.1632 (4)	0.0454 (5)	
C5	0.4197 (2)	0.41145 (7)	1.2349 (4)	0.0433 (5)	
H5A	0.4142	0.3960	1.3688	0.052*	
C6	0.5656 (3)	0.40510 (7)	1.0969 (3)	0.0384 (4)	
C7	0.8262 (3)	0.37562 (7)	0.9832 (3)	0.0373 (4)	
C8	0.9925 (2)	0.34762 (6)	0.9893 (3)	0.0374 (4)	
C9	1.0346 (3)	0.31950 (7)	1.1723 (3)	0.0422 (5)	
C10	1.1925 (3)	0.29284 (8)	1.1754 (4)	0.0485 (5)	
H10A	1.2205	0.2740	1.2959	0.058*	
C11	1.3085 (3)	0.29387 (8)	1.0030 (4)	0.0477 (5)	
H11A	1.4134	0.2756	1.0077	0.057*	
C12	1.2696 (2)	0.32199 (7)	0.8226 (4)	0.0437 (5)	
C13	1.1118 (3)	0.34856 (7)	0.8157 (4)	0.0425 (5)	
H13A	1.0850	0.3672	0.6942	0.051*	
C14	1.5246 (3)	0.29354 (9)	0.6280 (5)	0.0654 (7)	
H14A	1.5876	0.2998	0.4958	0.098*	
H14B	1.4815	0.2603	0.6279	0.098*	
H14C	1.6038	0.2982	0.7482	0.098*	

### Atomic displacement parameters (Å^2^)

**Table:**

	*U*^11^	*U*^22^	*U*^33^	*U*^12^	*U*^13^	*U*^23^
S1	0.0447 (3)	0.0502 (3)	0.0418 (3)	0.0065 (2)	0.0063 (2)	0.0087 (3)
Cl1	0.0472 (3)	0.0645 (3)	0.0795 (4)	0.0118 (2)	0.0193 (3)	−0.0029 (3)
O1	0.0520 (9)	0.0678 (10)	0.0500 (9)	0.0074 (8)	0.0089 (8)	0.0207 (9)
O2	0.0586 (9)	0.0679 (11)	0.0590 (10)	0.0190 (8)	0.0183 (8)	0.0014 (9)
N1	0.0387 (8)	0.0426 (9)	0.0414 (9)	0.0025 (7)	0.0023 (8)	0.0032 (8)
C1	0.0405 (10)	0.0387 (10)	0.0450 (12)	−0.0017 (8)	0.0034 (9)	−0.0009 (9)
C2	0.0443 (10)	0.0467 (11)	0.0513 (12)	0.0031 (9)	−0.0012 (10)	0.0070 (11)
C3	0.0425 (11)	0.0429 (12)	0.0628 (14)	0.0075 (9)	−0.0046 (10)	0.0014 (11)
C4	0.0360 (10)	0.0419 (11)	0.0582 (13)	0.0014 (8)	0.0063 (9)	−0.0064 (10)
C5	0.0417 (10)	0.0422 (11)	0.0460 (12)	0.0003 (8)	0.0064 (10)	−0.0005 (9)
C6	0.0372 (9)	0.0356 (9)	0.0425 (11)	−0.0008 (8)	−0.0009 (8)	−0.0019 (9)
C7	0.0382 (9)	0.0368 (10)	0.0370 (10)	−0.0017 (8)	−0.0007 (8)	0.0016 (8)
C8	0.0377 (9)	0.0341 (9)	0.0405 (10)	−0.0001 (8)	−0.0012 (8)	−0.0028 (8)
C9	0.0397 (10)	0.0424 (11)	0.0445 (11)	−0.0049 (8)	0.0005 (9)	0.0014 (9)
C10	0.0433 (11)	0.0476 (12)	0.0547 (13)	0.0013 (9)	−0.0056 (10)	0.0131 (10)
C11	0.0377 (10)	0.0436 (13)	0.0618 (14)	0.0065 (9)	−0.0022 (10)	−0.0007 (10)
C12	0.0421 (10)	0.0409 (10)	0.0482 (13)	0.0013 (8)	0.0067 (9)	−0.0051 (9)
C13	0.0457 (10)	0.0423 (10)	0.0395 (12)	0.0042 (8)	0.0006 (8)	0.0003 (9)
C14	0.0486 (13)	0.0615 (14)	0.0862 (19)	0.0091 (11)	0.0176 (14)	−0.0097 (14)

### Geometric parameters (Å, º)

**Table:**

S1—C1	1.7364 (19)	C5—C6	1.397 (3)
S1—C7	1.755 (2)	C5—H5A	0.9300
Cl1—C4	1.749 (2)	C7—C8	1.460 (3)
O1—C9	1.357 (3)	C8—C13	1.397 (3)
O1—H1A	0.80 (3)	C8—C9	1.403 (3)
O2—C12	1.370 (3)	C9—C10	1.387 (3)
O2—C14	1.416 (3)	C10—C11	1.376 (3)
N1—C7	1.307 (3)	C10—H10A	0.9300
N1—C6	1.382 (2)	C11—C12	1.385 (3)
C1—C2	1.393 (3)	C11—H11A	0.9300
C1—C6	1.401 (3)	C12—C13	1.386 (3)
C2—C3	1.386 (3)	C13—H13A	0.9300
C2—H2A	0.9300	C14—H14A	0.9600
C3—C4	1.380 (3)	C14—H14B	0.9600
C3—H3A	0.9300	C14—H14C	0.9600
C4—C5	1.373 (3)		
			
C1—S1—C7	89.24 (10)	C13—C8—C9	119.16 (17)
C9—O1—H1A	105 (2)	C13—C8—C7	121.04 (18)
C12—O2—C14	117.9 (2)	C9—C8—C7	119.80 (17)
C7—N1—C6	111.61 (17)	O1—C9—C10	117.69 (19)
C2—C1—C6	120.9 (2)	O1—C9—C8	123.10 (18)
C2—C1—S1	129.54 (18)	C10—C9—C8	119.20 (19)
C6—C1—S1	109.53 (15)	C11—C10—C9	121.1 (2)
C3—C2—C1	117.9 (2)	C11—C10—H10A	119.4
C3—C2—H2A	121.0	C9—C10—H10A	119.4
C1—C2—H2A	121.0	C10—C11—C12	120.25 (18)
C4—C3—C2	120.19 (19)	C10—C11—H11A	119.9
C4—C3—H3A	119.9	C12—C11—H11A	119.9
C2—C3—H3A	119.9	O2—C12—C11	124.51 (18)
C5—C4—C3	123.4 (2)	O2—C12—C13	116.0 (2)
C5—C4—Cl1	118.03 (19)	C11—C12—C13	119.5 (2)
C3—C4—Cl1	118.56 (17)	C12—C13—C8	120.8 (2)
C4—C5—C6	116.8 (2)	C12—C13—H13A	119.6
C4—C5—H5A	121.6	C8—C13—H13A	119.6
C6—C5—H5A	121.6	O2—C14—H14A	109.5
N1—C6—C5	124.37 (19)	O2—C14—H14B	109.5
N1—C6—C1	114.83 (18)	H14A—C14—H14B	109.5
C5—C6—C1	120.79 (18)	O2—C14—H14C	109.5
N1—C7—C8	122.89 (18)	H14A—C14—H14C	109.5
N1—C7—S1	114.79 (15)	H14B—C14—H14C	109.5
C8—C7—S1	122.31 (15)		
			
C7—S1—C1—C2	−178.3 (2)	C1—S1—C7—C8	−179.84 (17)
C7—S1—C1—C6	0.67 (15)	N1—C7—C8—C13	179.49 (19)
C6—C1—C2—C3	0.2 (3)	S1—C7—C8—C13	−1.3 (3)
S1—C1—C2—C3	179.04 (17)	N1—C7—C8—C9	−0.9 (3)
C1—C2—C3—C4	0.4 (3)	S1—C7—C8—C9	178.30 (15)
C2—C3—C4—C5	−0.9 (3)	C13—C8—C9—O1	−179.88 (19)
C2—C3—C4—Cl1	179.96 (17)	C7—C8—C9—O1	0.5 (3)
C3—C4—C5—C6	0.7 (3)	C13—C8—C9—C10	−0.8 (3)
Cl1—C4—C5—C6	179.85 (15)	C7—C8—C9—C10	179.61 (19)
C7—N1—C6—C5	178.95 (19)	O1—C9—C10—C11	179.6 (2)
C7—N1—C6—C1	0.3 (2)	C8—C9—C10—C11	0.5 (3)
C4—C5—C6—N1	−178.67 (19)	C9—C10—C11—C12	0.3 (3)
C4—C5—C6—C1	−0.1 (3)	C14—O2—C12—C11	11.7 (3)
C2—C1—C6—N1	178.37 (18)	C14—O2—C12—C13	−169.1 (2)
S1—C1—C6—N1	−0.7 (2)	C10—C11—C12—O2	178.3 (2)
C2—C1—C6—C5	−0.4 (3)	C10—C11—C12—C13	−0.8 (3)
S1—C1—C6—C5	−179.42 (15)	O2—C12—C13—C8	−178.71 (18)
C6—N1—C7—C8	179.54 (18)	C11—C12—C13—C8	0.5 (3)
C6—N1—C7—S1	0.3 (2)	C9—C8—C13—C12	0.3 (3)
C1—S1—C7—N1	−0.57 (17)	C7—C8—C13—C12	179.88 (18)

### Hydrogen-bond geometry (Å, º)

**Table:**

*D*—H···*A*	*D*—H	H···*A*	*D*···*A*	*D*—H···*A*
O1—H1*A*···N1	0.80 (3)	1.87 (3)	2.612 (2)	154 (3)
C5—H5*A*···O2^i^	0.93	2.57	3.454 (3)	159

Symmetry code: (i) *x*−1, *y*, *z*+1.
